# Performance Verification of Autonomous Driving LiDAR Sensors under Rainfall Conditions in Darkroom

**DOI:** 10.3390/s24010014

**Published:** 2023-12-19

**Authors:** Jaeryun Choe, Hyunwoo Cho, Yoonseok Chung

**Affiliations:** Transportation Safety Unit, Construction Division, Korea Conformity Laboratories (KCL), Nambusunhwan-ro 319-gil, Seocho-gu, Seoul 06711, Republic of Korea; choejr@kcl.re.kr (J.C.); hwcho@kcl.re.kr (H.C.)

**Keywords:** automotive LiDAR, darkroom, point cloud, rainfall, intensity

## Abstract

This research aims to assess the functionality of the VLP-32 LiDAR sensor, which serves as the principal sensor for object recognition in autonomous vehicles. The evaluation is conducted by simulating edge conditions the sensor might encounter in a controlled darkroom setting. Parameters for environmental conditions under examination encompass measurement distances ranging from 10 to 30 m, varying rainfall intensities (0, 20, 30, 40 mm/h), and different observation angles (0°, 30°, 60°). For the material aspects, the investigation incorporates reference materials, traffic signs, and road surfaces. Employing this diverse set of conditions, the study quantitatively assesses two critical performance metrics of LiDAR: intensity and NPC (number of point clouds). The results indicate a general decline in intensity as the measurement distance, rainfall intensity, and observation angles increase. Instances were identified where the sensor failed to record intensity for materials with low reflective properties. Concerning NPC, both the effective measurement area and recorded values demonstrated a decreasing trend with enlarging measurement distance and angles of observation. However, NPC metrics remained stable despite fluctuations in rainfall intensity.

## 1. Introduction

Road transportation is among the most prevalent forms of conveyance. A report by the World Health Organization reveals that road accidents claim approximately 1.3 million lives and cause injuries to nearly 50 million individuals annually. Further, research by the U.S. National Highway Traffic Safety Administration attributes 94% to 96% of vehicular accidents to driver negligence and incorrect interpretation of road signs [1]. To ensure the safe operation of autonomous vehicles, perception sensors such as LiDAR, cameras, and radars must be utilized to obtain real-time information regarding the location, shape, distance, speed, and direction of obstacles. Deep learning-based algorithms must also accurately predict object movement and interactions over time. Autonomous driving constitutes a continuous adaptive process that considers real-time location data and the movement patterns of pedestrians and adjacent vehicles. Improvements in the performance of perception sensors and deep learning algorithm technologies are critical for increasing the reliability of autonomous driving systems. It is also vital to adapt the current traffic infrastructure, primarily designed based on human visual criteria, to be compatible with the sensory capabilities of autonomous vehicles. Accurate traffic sign recognition is particularly crucial for the safe operation of these vehicles [2,3,4]. In Austria, lane-keeping assist systems have been found to prevent 17% of fatal traffic accidents, indicating potential for further safety enhancements [5]. A separate study focusing on the impact of forward collision warning (FCW) and lane departure warning (LDW) systems concluded that these technologies could prevent up to 69% of driver injuries in rear-end collisions. Such findings emphasize the importance of expanding the range of sensors employed in vehicles for enhancing road safety.

While all components of autonomous driving technology—including perception, decision-making, and control mechanisms—are integral to its function, several accidents have been attributed to sensor misperception. Such cases involve autonomous vehicles mistaking a clear blue sky for the side of a white trailer, causing a collision, colliding with a median due to impaired sensor performance in strong backlight conditions, and failing to detect a jaywalking pedestrian during nighttime. These incidents underscore the risks associated with overreliance on advanced driver assistance systems (ADAS). Based on accident reports, many drivers either neglected to monitor the vehicle due to trust in the ADAS or encountered accidents due to the technical limitations of the vehicle’s sensory apparatus [6].

Autonomous vehicles are typically classified according to the six levels of technology delineated by the Society of Automotive Engineers. Research and development efforts are underway to attain the pinnacle of Level 5 autonomy. Although these systems operate reliably under optimal road conditions, their performance can be compromised under challenging circumstances that degrade the functionality of on-board perception sensors, such as in adverse weather conditions. For example, while autonomous vehicles display consistent operational performance under normal weather conditions, research has indicated a decline in efficacy under severe conditions like intense nocturnal rainfall [7]. Further, unfavorable weather conditions can impair the functionality of key perception sensors, including cameras and LiDAR, thereby hindering the seamless deployment of autonomous driving technologies [8].

Recent initiatives have been undertaken to standardize testing methods for automotive LiDAR, a critical component of autonomous driving systems. The commencement of work for ISO/TC22/SC32 marks a significant step in this direction [9]. This Working Group, which includes prominent automotive industry companies such as Hesai, Denso, Bosch, Sony, Nissan, and ZF, aims to develop international standards for automotive LiDAR. Once formalized, these standards will streamline the testing procedures for evaluating LiDAR performance, reducing the likelihood of object misrecognition.

In parallel, the North American standardization body, UL, is formulating LiDAR standards through UL4700 [10]. This standard encompasses a range of evaluation criteria, including mechanical, electrical, laser, network, and stability parameters. Within the purview of LiDAR’s functionalities, the standard addresses the “Detection Probability” item, which considers variables like low point clouds, low resolution, measurement distance, field of view (FOV), and range accuracy. It also includes “Environmental Constraints Issues”, which necessitate risk analysis and mitigation technologies to account for challenges posed by adverse weather conditions such as heavy rain, snow, hail, and fog.

Furthermore, the Institute of Electrical and Electronics Engineers (IEEE) Automated Vehicles Standards Committee (VT/AVSC) is in the process of establishing the IEEE P2936 standard [11]. This standard will outline evaluation methods for LiDAR, specifically focusing on point cloud data. It will include key evaluation metrics such as region of interest accuracy, precision, resolution, and reflective performance detection rate.

In addition to these emerging standards, existing regulations aim to enhance the safety and reliability of autonomous driving systems. For instance, the ISO 26262 standard, established in 2018, addresses the functional safety of road vehicles by mitigating risks arising from malfunctions in their electrical and electronic systems [12]. ISO 21434, introduced in 2021, offers solutions for countering cyber-attacks or potential vulnerabilities in vehicle electrical and electronic control systems, hardware, software components, and interfaces [13]. Most recently, ISO 21448 was formalized in 2022 to manage the risks associated with sensor or system performance limitations, drivers’ unexpected misuse, and unforeseen road conditions change when autonomous vehicles are operational [14].

## 2. Literature Review

Challenging environmental conditions such as rain, snow, and fog often lead to performance limitations in perception sensors like LiDAR. Many studies have been conducted to evaluate the impact of these adverse conditions on autonomous vehicle perception systems. There are two reasons for reducing the performance of LiDAR during rainfall: First, the number of point clouds (NPC) obtained is significantly reduced as the laser pulse reflects off objects due to collisions with raindrops. Second, the accuracy of the distance to the object is reduced because the laser pulse collides with the raindrop and returns before reaching the object. Weather environments other than rainfall, such as fog and snow, also degrade LiDAR performance for similar reasons [15,16].

One notable study by Heinzler et al. (2019) focused on heavy rain and fog environments to assess the efficacy of LiDAR sensors. Their experimental setup involved 55 mm/h rainfall conditions and varied visibility distances between 20 and 60 m, segmented into 10-m intervals within a controlled chamber. The study observed that the number of point clouds diminished under rainy conditions compared to dry scenarios. Additionally, in foggy conditions, atmospheric particles scattered laser outputs, thereby reducing the object recognition rate of LiDAR [17]. Rivero et al. (2020) conducted a comprehensive outdoor study utilizing mechanical and solid-state LiDAR sensors. These sensors were mounted on building rooftops and focused on an asphalt parking lot surface. This investigation spanned nine months and incorporated weather conditions, including clear skies, rain, fog, and snow. The study’s strength lies in its systematic data collection under actual environmental conditions, compared with data gathered in a controlled setting, thereby minimizing measurement errors [18]. Similarly, Montalban et al. (2021) conducted an indoor quantitative performance assessment using five different types of LiDAR sensors. These were categorized based on various parameters: the measurement methodology employed (mechanical or MEMS type), the number of channels (24, 32, 128 channels), and the operating wavelength bands (850, 905, 1550 nm). The study considered rainfall intensities ranging from 20 to 120 mm/h and established a visibility range based on fog density ranging from 10 to 80 m. These research endeavors highlight the significant impact of adverse weather conditions on the performance of LiDAR sensors, underscoring the need for further investigations and improvements in this area. The AEye 4SightM sensor, which employs the MEMS method, displayed remarkable resilience against varying rainfall intensities, maintaining a stable number of point clouds. This performance is ascribed to the sensor’s high laser output value in the 1550 nm wavelength band, which allows for enhanced laser penetration and superior target detection. While indoor chambers with artificially generated weather conditions offer a quantitatively reproducible environment, discrepancies may exist when compared to natural weather phenomena, necessitating caution in applying such research findings [19]. LiDAR uses laser pulses reflected from an object, affecting its performance by the object’s material and the reflective surface’s color. Additionally, LiDAR performance based on color appears better when the color is white rather than a dark achromatic color [20]. A study by Kim et al. (2012) observed that the maximum measurable distance for targets varies among LiDAR manufacturers and can range from 120 to 250 m for highly reflective materials. However, for targets composed of materials with approximately 10% reflectivity, the measurable distance diminishes to around 50 m [21].

Jokela et al. (2019) evaluated how the performance of LiDAR sensors is influenced by fog density in indoor settings. The study found that as fog density increased and the measurement distance expanded, the efficiency of LiDAR sensors declined. Additionally, outdoor tests considered the performance of LiDAR units mounted on both the roofs and bumpers of vehicles, especially under conditions involving sub-zero temperatures and reduced visibility due to snow particles scattered during driving. These particles, generated by leading and the LiDAR-equipped vehicles, considerably hamper visibility [22]. Filgueira et al. (2017) conducted an experiment measuring surfaces of various materials commonly found alongside roads, such as metal billboards, concrete walls, glass walls, traffic signs with retro-reflective surfaces, asphalt pavement, and retro-reflective pavement. The LiDAR-to-target measurement distance was maintained within 100 m. This study adds a further dimension to our understanding of how material reflectivity may interact with LiDAR sensors under different environmental conditions. Collectively, these studies shed light on the multifaceted challenges facing LiDAR sensors in adverse weather and offer avenues for further research and development. In the study conducted by Filgueira et al. (2017), continuous measurements were made on various targets during rainfall to analyze both the absolute reflectivity values based on material composition and the rate of reflectivity reduction due to precipitation. The study revealed that facilities composed of highly reflective, retro-reflective materials experienced a steep decline in reflectivity during rainfall. Conversely, materials with lower reflectivity—such as concrete, asphalt, and glass—manifested a more gradual reduction in reflectivity levels [23].

Linnhoff et al. (2022) engaged in a six-month outdoor investigation to scrutinize the impact of varying weather conditions, including fog, rain, and snow, on LiDAR sensors. The experimental design incorporated three distinct types of LiDAR sensors, in conjunction with fog and rainfall sensors, to measure a reference material (RM) positioned 50 m away. The results indicated that as the intensity of fog and rain escalated, the performance of the LiDAR sensors correspondingly deteriorated. Specifically, the study found that in foggy conditions, the scattering of light due to atmospheric particles adversely affected the LiDAR’s ability to detect point clouds. During rainfalls, sensing efficacy began to decline at a rainfall intensity of 10 mm/h, and at 50 mm/h, target detection was essentially nullified. However, it was observed that varying levels of snowfall did not lead to a marked decline in the LiDAR’s sensing capabilities [24]. Recently, many researchers have been researching to improve the recognition rate of autonomous driving by converging LiDAR and image sensors. When recognizing objects by fusing LiDAR and images, recognition performance is superior to when using images alone. Therefore, convergence technology of multiple sensors is essential to improve the driving safety of autonomous vehicles [25,26].

## 3. Methodology

This study employed the Velodyne LiDAR VLP-32, which boasts a maximum measurement distance of 200 m, an accuracy margin of ±3 cm, a horizontal FoV spanning 360 degrees, and a vertical FoV of 40 degrees, as described in Table 1. The device’s horizontal resolution ranged between 0.1 and 0.4°, its minimum vertical resolution stood at 0.33°, and it offered a frame rate that could be configured between 5 and 20 frames per second. Operating in the 905 nm laser wavelength band, the Velodyne VLP-32 was calibrated to collect data in 10-frame increments per second. Subsequently, the average intensity and number of point cloud (NPC) values were assessed for 100 frames, spanning 10 s.

**Figure 1:**
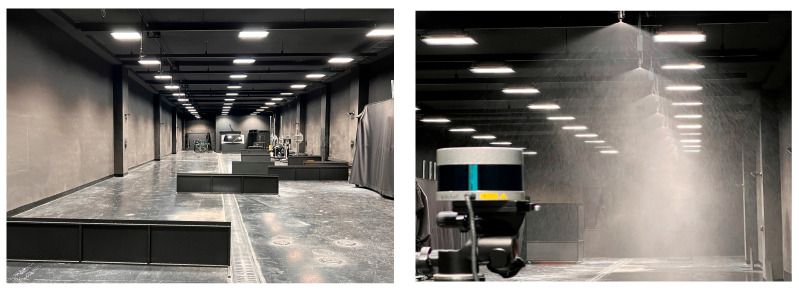
Overview of darkroom and rainfall experiment.

Korea Conformity Laboratories (KCL) operates a specialized weather reproduction chamber measuring 35 m in length, 6.5 m in width, and 4 m in height, as shown in Figure 1. The internal surfaces of this chamber are coated with a non-reflective paint to minimize light scattering, rendering the environment conducive for assessing the visibility of road traffic infrastructure and for vehicle lighting system evaluations. The chamber is equipped with a rainfall simulation device capable of replicating rainfall intensities that range from 20 to 40 mm/h, incremented in steps of 5 mm/h. Furthermore, the chamber’s lighting system can emulate various natural lighting conditions, including daytime, nighttime, sunrise, and sunset. It offers a luminance spectrum ranging from 2000 to 1 lx, a color temperature scale of 2700 to 6500 K, and a color rendering index (CRI) that exceeds 97.

**Figure 2:**
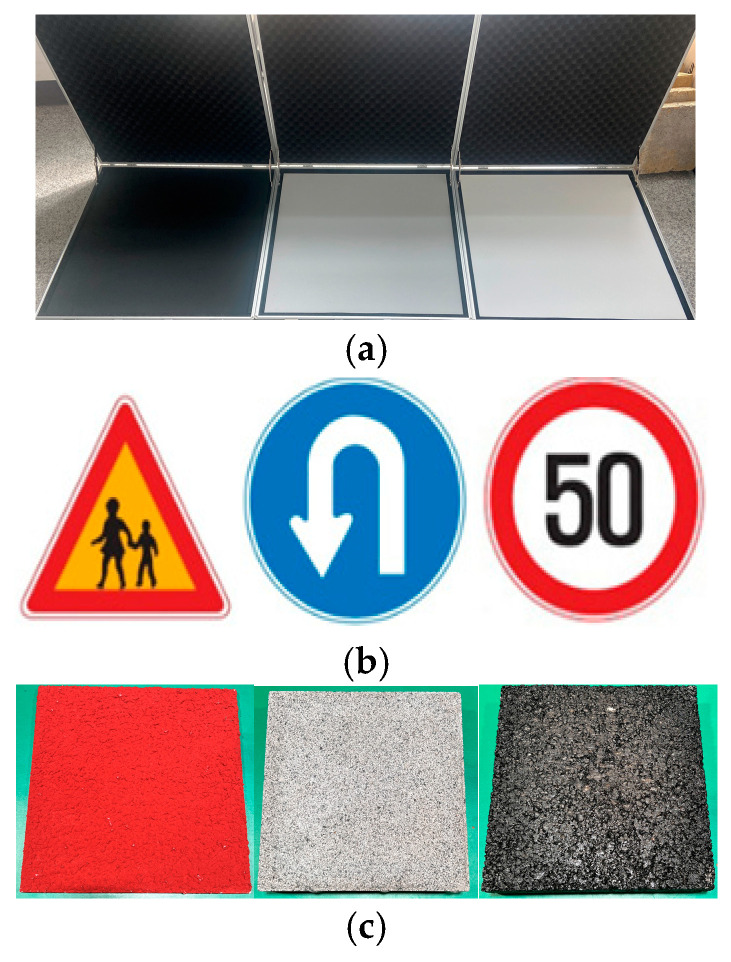
Materials employed for testing. (**a**) Reference Materials (RM 5%, 50%, 95%); (**b**) Traffic signs (school zone, U-turn, speed limit); (**c**) Pavement materials (skid resistance, concrete, asphalt).

**Figure 3:**
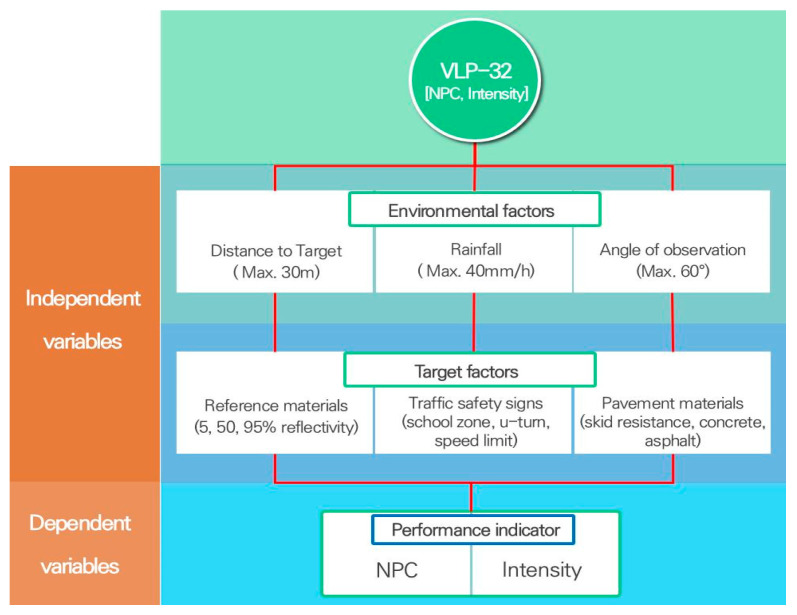
A flow chart for the overall test procedure.

To ascertain the validity of crucial LiDAR performance indicators, precisely intensity and NPC, the experimental setup accounted for variables such as measurement distance, precipitation intensity, and observational angle. The experiments incorporated various facility factors, including reference materials and three categories of traffic safety signs: school zone signs, U-turn signs, and speed limit signs, as shown in Figure 2. In addition, three different road surface materials—skid-resistant pavement, concrete pavement, and asphalt pavement—were examined. Integrated experimental scenarios considering these environmental and target factors are described in Table 2. In addition, the flow chart for the overall test procedure considering independent and dependent variables in this study is sequentially explained in Figure 3.

While LiDAR manufacturers furnish basic device specifications, the experiments employed reference materials to validate one of LiDAR’s critical performance parameters: intensity. These reference materials benchmarked the reflective properties of various road traffic facilities against LiDAR-generated ’reflectivity (intensity) metrics. Specifically, these reference reflectance targets, each measuring 0.5 by 0.5 m, are produced by Labsphere. They are calibrated by both the Physikalisch-Technische Bundesanstalt (PTB) in Germany and the National Institute of Standards and Technology (NIST) in the United States, ensuring that they consistently manifest reflective values within a surface reflectance tolerance of ±1% [27]. Incorporating these meticulously calibrated reference materials enhances the rigor and reliability of the evaluation, thereby contributing valuable data for understanding the performance metrics of LiDAR sensors under varied environmental conditions.

## 4. Results and Discussion

**Figure 4:**
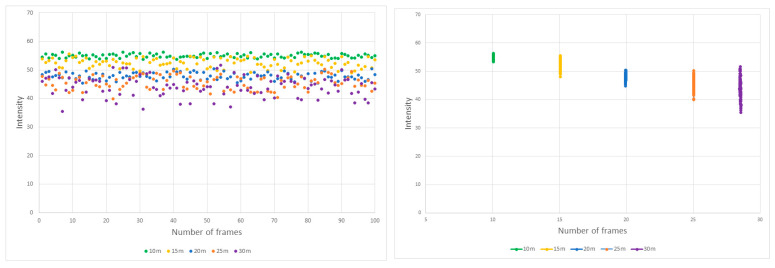
Intensity measurements for RM (50%) over a 10-s duration.

In this investigation, the Velodyne LiDAR VLP-32 was utilized to record intensity values as a function of measurement distance. The device was configured to collect data at ten frames per second, and the average intensity value was computed based on a 10-s data capture interval. All graphical representations in this study encapsulate average intensity and NPC values across 100 frames. Additionally, the error bars in these graphs signify the standard deviation (±σ). For example, Figure 4 delineates the intensity values for reference material RM50 across various measurement distances. Corresponding average values and standard deviations are tabulated in Table 3. At a measurement distance of 10 m, the intensity was registered at 54.9 units. Upon increasing the measurement distance to 30 m, the intensity diminished to 44.8 units, constituting approximately 81% of the initial value. Concurrently, as the measurement distance was extended, average intensity values exhibited a downward trend, while the standard deviation demonstrated an incline.

**Figure 5:**
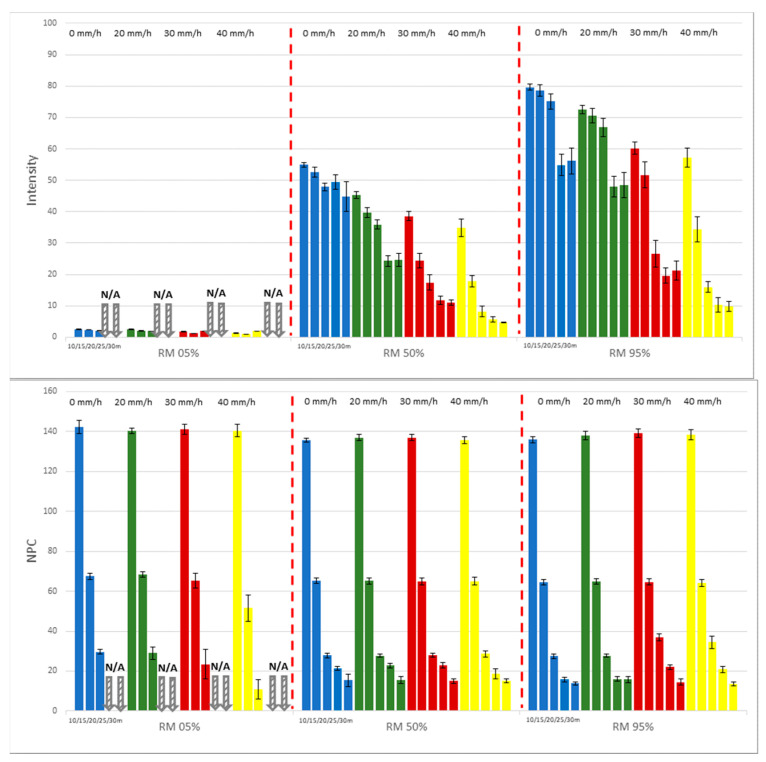
Variation in intensity and NPC values for RM (5%, 50%, 95%) with respect to distance and rainfall.

To evaluate key performance indicators of LiDAR—namely, intensity and NPC—reference materials designated as RM (5%, 50%, 95%) were employed. These measurements were taken across five distance increments (10, 15, 20, 25, 30 m) and four gradations of rainfall intensity (0, 20, 30, 40 mm/h). Notably, at a distance of 10 m and without rainfall, intensity measurements for RM 95% and RM 5% were recorded as 82.6 and 2.6 units, respectively. These figures are notably lower than their designated reflectivity percentages. Conversely, the intensity measurement for RM 50% was 54.9, a value exceeding its reflectivity percentage. Figure 5 illustrates that as both measurement distance and rainfall intensity escalate, there is a general decline in intensity values, irrespective of the type of reference material in use. In contrast, NPC values demonstrate a decreasing pattern with an increase in distance but remain impervious to variations in rainfall intensity.

Upon analysis of the data for reference material RM 95%, which possesses the highest reflectivity, several observations can be made. The intensity value, initially measured at 82.6 units at a proximal distance of 10 m under clear atmospheric conditions, diminishes to 56.1 units (67.9% of the initial value) when the measurement distance is extended to 30 m. A further reduction to 57.1 units (69.1% of the initial value) occurs at a peak rainfall intensity of 40 mm/h. However, when both variables—maximum measurement distance of 30 m and maximum rainfall intensity of 40 mm/h—are combined, the intensity value drastically declines to 9.8 units, accounting for merely 11.8% of the original measurement. For RM 5%, distinguished by its minimal reflectivity, intensity, and NPC values, it is not recorded at measurement distances exceeding 25 m, irrespective of the level of rainfall intensity. While NPC values remain constant despite increasing levels of rainfall intensity, a decline is observed as the measurement distance is elongated. This decreasing trend in NPC values is ascribed to the contraction of the effective area that the LiDAR is capable of scanning at its inherent horizontal and vertical resolutions. As the measurement distance expands, this effective area diminishes, leading to a consequential decrease in NPC values.

Traffic signs are predominantly situated alongside roadways; thus, LiDAR sensors affixed to autonomous vehicles generally possess a fixed horizontal angle of observation. To simulate these conditions, experiments were conducted with varying angles of observation set at three levels: 0°, 30°, and 60°. These tests were executed using a reference material with a 50% reflectivity and dimensions of 0.5 × 0.5 m. The performance indicators of LiDAR, specifically intensity and NPC values, were assessed based on measurement distance and rainfall intensity.

**Figure 6:**
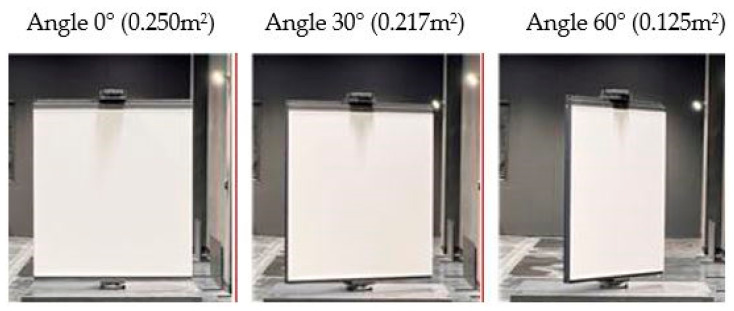
Reduction in the effective area according to the angle of observation.

As illustrated in Figure 6, at a 0° angle of observation, the effective measurement area is 0.250 m^2^. This area decreases by approximately 50% to 0.125 m^2^ when the angle of observation is elevated to 60°. A corresponding decrease in NPC values is observed as the angle increases, owing to the reduction in the effective measurement area. Under the most unfavorable conditions—namely, a 60° angle of observation, a 30 m measurement distance, and a 40 mm/h rainfall intensity—neither intensity nor NPC values were recorded.

**Figure 7:**
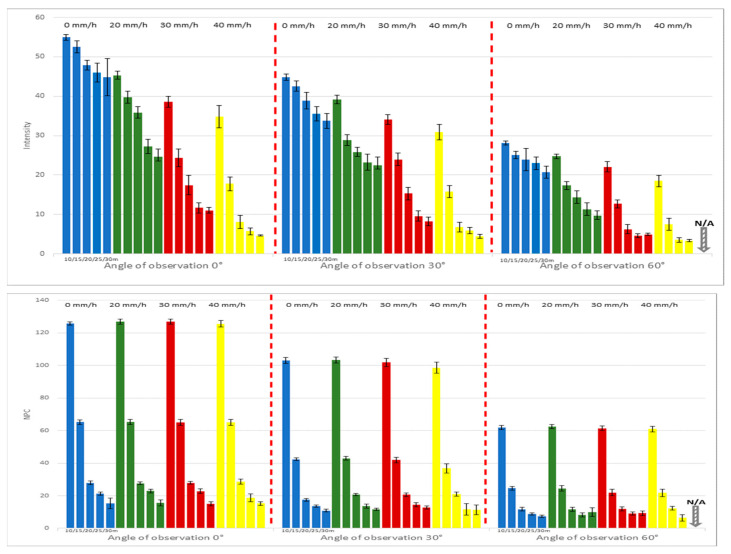
The intensity and NPC values for RM (50%) are affected by the angle of observation.

Figure 7 and Table 4 further corroborate that an increasing measurement distance and rainfall intensity correspond to diminishing intensity values, and this trend is consistent across all observed angles (0°, 30°, 60°). Specifically, at a 0° angle of observation, the maximum intensity value registered was 54.9, while the minimum was 4.7, constituting approximately 8.5% of the maximum. At a 30° angle, the maximum and minimum intensity values were 44.9 and 4.5, respectively, with the minimum representing around 10.0% of the maximum. For an angle of 60°, only a maximum intensity value of 28.1 was observed; a minimum value was not recorded.

Consequently, it is imperative to recognize that, in addition to measurement distance and rainfall intensity, the angle of observation significantly influences the efficacy of LiDAR systems.

**Figure 8:**
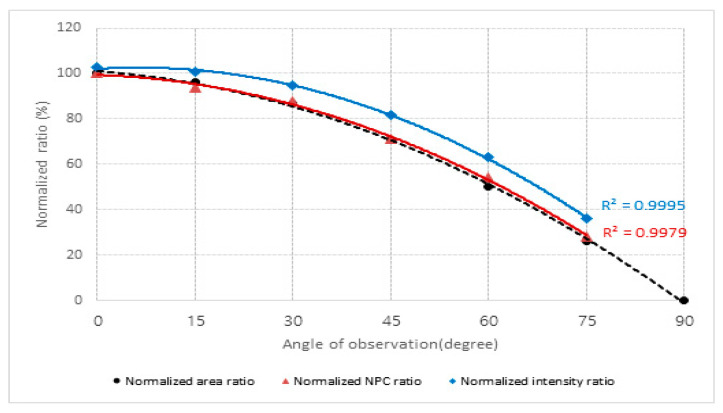
Comparative analysis of intensity and NPC ratios across different angles of observation.

Owing to the substantial influence of the angle of observation on LiDAR performance, particularly when the alignment between LiDAR sensors and traffic facilities deviates from a linear configuration, supplementary experiments were designed to scrutinize the relationship between the angle of observation and key performance metrics of LiDAR: intensity and NPC. The intensity and NPC values were acquired at a 10-m distance using reference material RM 50% at multiple angles of observation (0, 15, 30, 40, 60, 75°). The effective area corresponding to each angle of observation was calculated theoretically. Subsequently, normalization procedures were implemented by dividing these calculated areas by the foundational area corresponding to a 0° angle of observation. In a parallel manner, intensity and NPC values were ascertained for diverse angles, and normalization was conducted using the peak intensity and NPC values at a 0° angle of observation as reference points. These results are elaborated in Figure 8 and Table 5.

As the angle of observation enlarges, the normalized NPC ratio diminishes in a fashion that mirrors the reduction in the normalized area ratio. The margin of error for each angle of observation resides within the bounds of 0.9–8.8%. Thus, if the NPC metric for a particular LiDAR model (VLP-32) is known at a 0° angle of observation, it becomes feasible to forecast with relative accuracy the descending trajectory of the NPC values as the effective area changes with varying angles of observation. Similarly, as the angle of observation escalates, the normalized intensity ratio exhibits a propensity for overestimation when compared to the normalized area ratio, and the margin of error for each angle of observation ranges between 0.9–35.8%.

**Figure 9:**
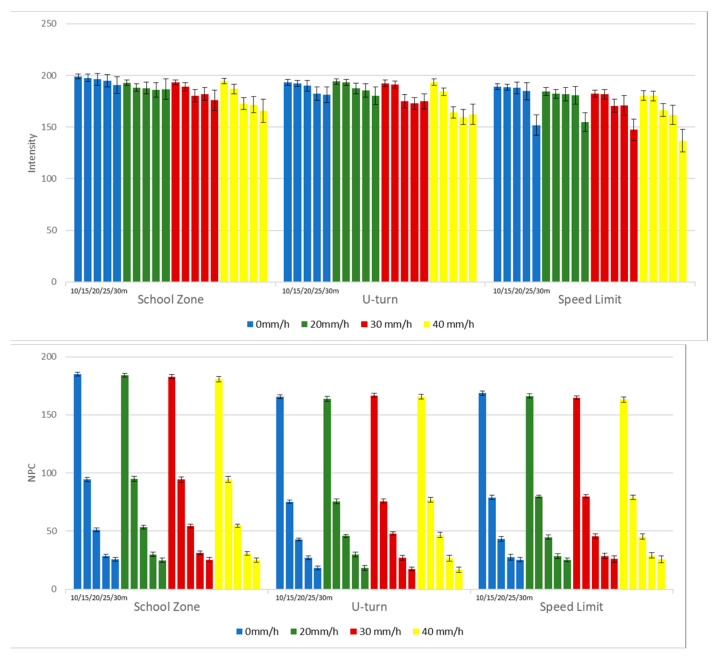
Intensities and NPCs of various traffic signs.

In light of experimental results garnered from utilizing RMs, actual traffic signs were employed to gauge key LiDAR performance metrics—intensity and NPC—at diversified distances (10, 15, 20, 25, 30 m) and varying levels of rainfall intensity (0, 20, 30, 40 mm/h). The types of traffic signs selected for scrutiny included school zones, U-turns, and speed limit indicators. As delineated in Figure 9, the recorded intensity values spanned from 165 to 198 for school zone signs, 159 to 194 for U-turn signs, and 136 to 189 for speed limit signs. The attenuation in intensity values with ascending measurement distance and increasing rainfall was less pronounced when compared to that observed with RMs. This can be attributed to the high-reflectivity sheeting material that constitutes the traffic signs, rendering them relatively impervious to variations in measurement distance and rainfall intensity. Consistent with prior observations, NPC values decline as the measurement distance enlarges, although remaining unresponsive to changes in rainfall intensity. Elevated NPC values for school zone signs compared to U-turn and speed limit signs can be reasonably ascribed to the approximately 7% greater effective area of school zone signs.

**Figure 10:**
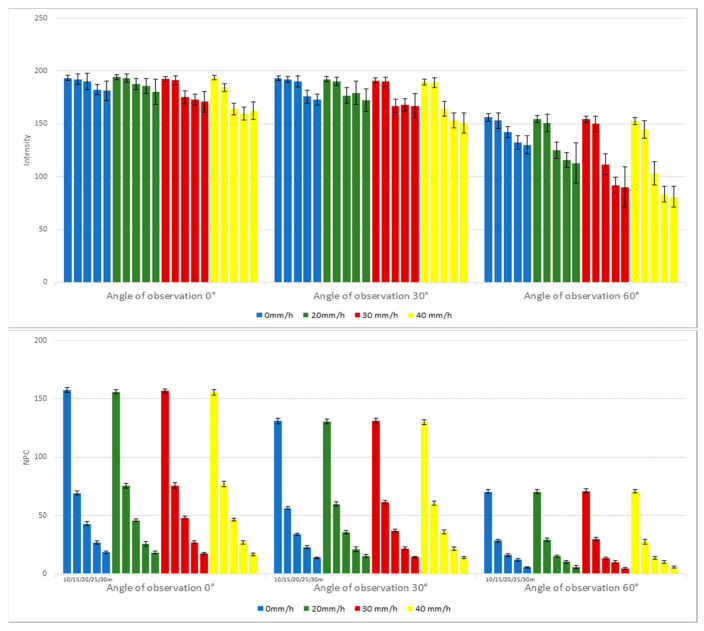
Intensities and NPCs of U-turn as functions of angle of observation.

Considering the robust reflectivity of the traffic signs, both measurement distance and rainfall intensity had a marginal impact on the performance indicators. An isolated experiment focusing solely on U-turn signs was devised to further elucidate the role of the angle of observation, another pivotal factor affecting LiDAR’s efficacy. As detailed in Figure 10 and Table 6, at a 0° angle of observation, the upper and lower intensity values were 193.3 and 162.4, constituting approximately 84.0% of the maximum. When the angle of observation was adjusted to 30°, the lowest recorded intensity value was 151.0, marking a 78.1% reduction relative to the peak value. At a 60° angle of observation, the minimal intensity value precipitously dwindled to 81.0, equating to roughly 51.9% of the highest recorded value. Consistent with previous findings, a rising angle of observation results in the contraction of the effective area, thereby causing a concomitant diminution in NPC values. Notably, rainfall intensity continued to demonstrate no discernible influence on these metrics.

**Figure 11:**
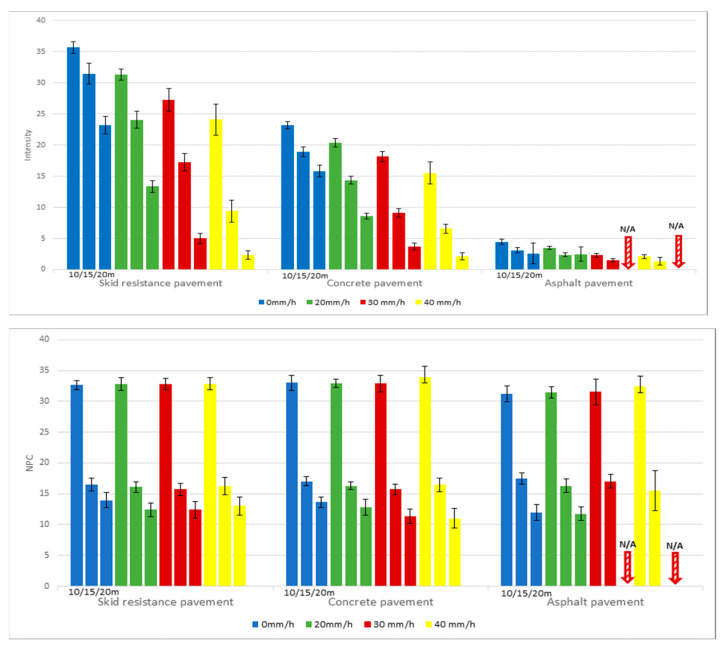
Intensity and NPC values across different types of pavement.

The performance indicators for LiDAR—intensity and NPC—were quantified for various types of road pavement (0.3 × 0.3 m) commonly encountered by autonomous vehicles across different distances and levels of rainfall intensity. Owing to the intrinsically low reflectivity of these road materials, neither intensity nor NPC values were recorded at distances exceeding 25 m, irrespective of the level of precipitation. Specifically, for asphalt pavement, which exhibits the least reflectivity among the materials studied, the absence of recorded intensity and NPC values was noted at distances of 20 m or beyond under rainfall intensities of 30 mm/h or greater. As indicated in Figure 11, the captured intensity values for skid-resistant pavement fluctuated between 2 and 35, while those for concrete pavement varied from 2 to 23. This considerable variance underscores the influence of measurement distance and rainfall intensity on the recorded values. In the instance of asphalt pavement, the obtained values were notably minimal, spanning merely from 1 to 4.

## 5. Conclusions

This research assessed critical performance indicators for LiDAR technology—precisely intensity and NPC—to evaluate the efficacy of an autonomous vehicle’s LiDAR sensor (VLP-32). This evaluation encompassed a range of environmental variables (measurement distance, rainfall intensity, and angle of observation) and material attributes of the observed facilities (RM, traffic signs, and road pavements). The key findings are summarized as follows:

For the RM with the highest reflectivity in this study, an intensity value of 79.6 was recorded at a minimum measurement distance of 10 m under non-raining conditions. However, this value plummeted to 9.8 (11.8%) of the initial measurement under the most unfavorable conditions of a maximum measurement distance of 30 m and maximum precipitation of 40 mm/h. Additionally, for RM, which had the lowest reflectivity at 5%, no intensity or NPC values were recorded at distances exceeding 25 m, regardless of rainfall conditions. The maximum measurement distance of LiDAR sensors during autonomous driving is approximately 200 m, so NPC measurements for long-distance targets during rainfall are considered problematic.

When observations were made at a 0° angle, the highest recorded intensity value was 54.9, contrasting sharply with the minimum value of 4.7, constituting a reduction to 8.5% of the maximum. At a 60° angle of observation, the maximum intensity value was 28.1; however, a corresponding minimum value was not recorded. Therefore, it is crucial to consider the angle of observation as a significant variable influencing LiDAR performance alongside measurement distance and rainfall intensity.

When the angle of observation increases—from not being installed in a straight line between the LiDAR sensor and traffic signs—the normalized NPC ratio exhibited a decline analogous to that of the normalized area ratio. Consequently, if the NPC value for a specific LiDAR sensor (VLP-32) at a 0° angle of observation is known, it becomes feasible to predict, with reasonable accuracy, the decline in NPC due to alterations in the effective observational area at different angles.

For traffic signs, the intensity values exhibited a relatively modest rate of decline with increasing measurement distance and rainfall intensity, compared to RMs. This can be attributed to the highly reflective materials from which the traffic signs are made, rendering them less susceptible to variations in measurement distance and rainfall intensity. Similar to previous RM experiment results, the effective area decreases as the observation angle increases, and the NPC measurement value decreases accordingly. NPC values are affected by measurement distance and observation angle but are considered to not correlate with rainfall intensity.

Due to the inherently low reflective qualities of these road materials, neither intensity nor NPC values were recorded at distances exceeding 25 m, irrespective of the level of rainfall. Specifically, for asphalt pavement—the material with the lowest reflectivity—no intensity or NPC measurements were obtained at distances beyond 20 m when the rainfall intensity exceeded 30 mm/h.

This study analyzed the results of an experiment in a limited space called a dark room using a specific LiDAR sensor (VLP-32). Therefore, based on the results of this study, future research is needed to compare the performance of various types of LiDAR sensors and analyze the results collected from LiDAR sensors installed on autonomous vehicles.

## Figures and Tables

**Table 1:** Technical specifications of VLP-32.

Categories	Technical Specification
Sensors	Time of Flight distance measurement Layers: 32 Measurement range: ~200 m Range accuracy: up to ±3 cm Horizontal FoV: 360°, Vertical FoV: 40° Horizontal resolution: 0.1~0.4°, Vertical resolution: 0.33°(min) Rotation rate: 5~20 Hz Weight: 925 g
Laser	Wavelength: 905 nm
Output	Points per second (single return/dual return): ~600,000/~1,200,000

**Table 2:** Test Scenarios.

Categories	Items	Scenarios by Item				
Environmental Factors	Distance (m)	10	15	20	25	30
	Rainfall (mm/h)	0	20		30	40
	The angle of observation (°)	0		30		60
Target Factors	Materials	Reference Materials (5%, 50%, 95% reflectivity) Traffic signs (school zone, U-turn, speed limit) Pavement materials (skid resistance, concrete, asphalt)				

**Table 3:** Mean and standard deviation of measured intensities for RM (50%).

Categories	10 m	15 m	20 m	25 m	30 m
Average Intensity	54.9	52.5	47.8	45.5	44.8
Standard deviation (σ)	0.68	1.63	1.22	2.35	4.69

**Table 4:** Rate of intensity reduction for RM (50%) based on angle of observation.

Categories		Intensity (Intensity Reduction Rate, %)				
		10 m	15 m	20 m	25 m	30 m
0° angle of observation	0 mm/h	**54.9 (100.0)**	52.5 (95.6)	47.9 (87.1)	46.0 (83.7)	44.8 (81.6)
	40 mm/h	34.8 (63.4)	17.8 (32.3)	8.1 (14.8)	5.8 (10.5)	**4.7 (8.5)**
30° angle of observation	0 mm/h	**44.9 (100.0)**	42.6 (94.9)	38.8 (86.6)	35.5 (79.1)	33.70 (75.2)
	40 mm/h	30.9 (69.0)	15.8 (35.2)	6.8 (15.2)	5.9 (13.2)	**4.5 (10.0)**
60° angle of observation	0 mm/h	**28.1 (100.0)**	25.1 (89.2)	23.9 (85.1)	23.0 (81.7)	20.7 (73.5)
	40 mm/h	18.5 (65.8)	7.5 (26.8)	3.5 (12.6)	3.3 (11.8)	**N/A**

**Table 5:** Normalized intensity and NPC ratios as a function of angle of observation for RM (50%).

**The angle of observation (°)**	0	15	30	45	60	75
**Area (m^2^)**	0.25	0.241	0.217	0.177	0.125	0.065
**Normalized area (m^2^)**	100	96.4	86.8	70.8	50	26
**Normalized NPC ratio (%)**	100	93.8	87.6	71.1	54.4	28.3
**Normalized Intensity ratio (%)**	100	98.2	92.4	79.8	61.5	35.3

**Table 6:** Rate of intensity reduction for U-turn as a function of angle of observation.

Categories		Intensity (Intensity Reduction Rate, %)				
		10 m	15 m	20 m	25 m	30 m
0° angle of observation	0 mm/h	**193.3 (100.0**)	192.1 (99.4)	190.0 (98.3)	182.3 (94.3)	181.3 (93.8)
	40 mm/h	193.3 (99.9)	184.4 (95.4)	164.0 (84.9)	159.7 (82.6)	**162.4 (84.0)**
30° angle of observation	0 mm/h	**193.3 (100.0)**	192.0 (99.3)	190.0 (98.3)	175.7 (90.9)	173.0 (89.5)
	40 mm/h	189.6 (98.1)	188.7 (97.6)	164.3 (85.0)	153.2 (79.3)	**151.0 (78.1)**
60° angle of observation	0 mm/h	**156.1 (100.0)**	153.0 (98.0)	142.3 (91.2)	132.3 (84.7)	130.0 (83.3)
	40 mm/h	152.6 (97.7)	144.6 (92.7)	103.2 (66.1)	83.4 (53.4)	**81.0 (51.9)**

## Data Availability

Data are contained within the article.
